# Concurrent Resistance and Cardiorespiratory Training in Patients with Hypertrophic Cardiomyopathy: A Pilot Study

**DOI:** 10.3390/jcm13082324

**Published:** 2024-04-17

**Authors:** Adrián Bayonas-Ruiz, Francisca M. Muñoz-Franco, María Sabater-Molina, Ignacio Martínez-González-Moro, Juan Ramon Gimeno-Blanes, Bárbara Bonacasa

**Affiliations:** 1 Department of Physiology, Human Physiology Area, Sports Sciences Faculty, University of Murcia, C. Argentina, 19, 30720 San Javier, Murcia, Spain; adrian.bayonas@um.es; 2 Research Group of Physical Exercise and Human Performance, University of Murcia, 30720 San Javier, Murcia, Spain; igmartgm@um.es; 3 Cardiac Unit, General University Hospital Caravaca, 30400 Caravaca de la Cruz, Murcia, Spain; fca.mariamf@gmail.com; 4 Cardiogenetic Laboratory, Instituto Murciano de Investigación Biosanitaria (IMIB), 30120 El Palmar, Murcia, Spain; mariasm@um.es; 5 European Reference Network for Rare and Low Prevalence Complex Diseases of the Heart (ERN-Guard Heart), 1105 Amsterdam, The Netherlands; jgimeno@secardiologia.es; 6 Department of Legal and Forensic Medicine, Faculty of Medicine, Health Sciences Campus, Avda. Buenavista n°32, 30120 El Palmar, Murcia, Spain; 7 Department of Physiotherapy, Faculty of Medicine, Health Sciences Campus, Avda. Buenavista n°32, 30120 El Palmar, Murcia, Spain; 8 Inherited Cardiac Disease Unit (CSUR), Hospital Universitario Virgen de la Arrixaca, 30120 El Palmar, Murcia, Spain; 9 Department of Internal Medicine (Cardiology), University of Murcia, 30720 San Javier, Murcia, Spain

**Keywords:** exercise, CPET, intervention, prognosis, echocardiography, HCM

## Abstract

**Background:** Exercise training in patients with HCM has evidenced benefits on functional capacity, cardiac function, and a reversion of adverse cardiac remodeling. The objective of this study was to assess the effect of a concurrent resistance and cardiorespiratory training program on functional capacity, biochemical parameters, and echocardiographic variables in a pilot group. **Methods:** Two HCM patients were evaluated before and after 12 weeks of individualized concurrent training with two sessions/week. Pre- and post-training data were compared for each patient. Evaluations included a cardiopulmonary exercise test (CPET), body composition, echocardiography, electrocardiography, and blood analysis. **Results:** Training promoted an increase in functional capacity (+4 mL·kg^−1^·min^−1^), ventilatory thresholds, and other CPET-derived variables associated with a better prognosis and long-term survival. Muscular mass was augmented (0.8 and 1.2 kg), along with a mean increase of 62% in upper and lower body strength. Echocardiographic features demonstrated the maintenance of cardiac function with signs of positive left ventricular remodeling and an improvement in diastolic function. Blood analyses, including cardiac troponins and NT-proBNP, displayed uneven changes in each patient, but the values fell into normal ranges in both cases. **Conclusions:** The available data suggest a positive effect of concurrent resistance and cardiorespiratory training on patients’ functional capacity and cardiac function that may improve their functional class, quality of life, and long-term prognosis. The replication of this protocol in a larger cohort of patients is warranted to confirm these preliminary results.

## 1. Introduction

Hypertrophic cardiomyopathy (HCM) is the most prevalent cardiomyopathy (1:500) and is characterized by the presence of ventricular hypertrophy with a maximal wall thickness (MWT) of ≥15 mm in the absence of other primary causes [1,2]. Physical exercise in patients with HCM has emerged as an adjunctive treatment strategy, and its inclusion has been advocated in numerous theoretical proposals and reviews on potential benefits over the last decade [3,4]. Among these, researchers in this field are contemplating the possibility of improving functional capacity and effort tolerance, reversing cardiac remodeling, enhancing diastolic function, and improving end-systolic and end-diastolic volumes [5].

Regarding the impact of physical exercise, a recent meta-analysis conducted by our group demonstrated an improvement in VO_2_max of 4.33 mL/kg/min (95% CI: 0.20; 8.45), to a greater extent compared to other pharmacological and/or invasive treatment options, and an enhancement in New York Heart Association (NYHA) functional class [6]. Furthermore, none of the interventions resulted in serious adverse effects.

However, the practical implementation of physical exercise in HCM patients remains limited: starting from 2015, only five trials assessing the effects and safety of training have been published to date [7,8,9,10,11]. Despite most of them demonstrating global improvements with the program, significant heterogeneity in the methodology of the published studies limits the reproducibility of their findings. In particular, this regards the type of exercise, the definition of exercise load, thresholds and repetitions, the length of the training program, and methodology of the evaluation of the response to exercise. Most protocols were based on cardiorespiratory training and did not include strength exercises.

In light of the aspects discussed regarding the exercise methodology and its benefits, there is a need to optimize training protocols in HCM patients. Similarly, a more detailed description of the exercise is essential to facilitate its replication and use as an adjunct strategy in patient management. Advances in exercise physiology will be instrumental in optimizing variables for cardiorespiratory training (intensity based on VO_2_max and peak HR determined in CPET, duration, recovery, and frequency) and strength training (exercise selection, relative intensity -%RM-, effort grade/character, number of repetitions, daily and weekly volume, and rest between sets). This body of knowledge also encompasses the definition of “concurrent training”, which combines both resistance and cardiorespiratory training in a single program and/or training session [12].

Considering the latter, the objective of this study was to assess the effect of a concurrent resistance and cardiorespiratory training program on functional capacity, biochemical parameters, and echocardiographic variables in a pilot group of two HCM patients. In parallel, it is intended to evaluate the feasibility of the training protocol to replicate it in a larger cohort of patients. We hypothesized that a 12-week concurrent training regime would promote increases in functional capacity and strength without a detrimental effect on cardiac function.

## 2. Materials and Methods

### 2.1. Participants

The subjects of this study were two men aged 63 and 60 years with hypertrophic cardiomyopathy and a low level of physical activity, limited to their daily activities. Both patients underwent genetic testing of a gene panel associated with HCM, and this was negative. Patient 1 had obstructive HCM diagnosed 5 years before enrolling in the study, with a maximal left-ventricular outflow tract gradient of 90 mmHg after Valsalva maneuver. His medication included bisoprolol (2.5 mg/day), budesonide (320/9 mcg each 12 h when needed) for chronic obstructive pulmonary disease (COPD) potentially derived from a history of smoking habit, and levothyroxine (75 mcg/day). He had no previous major adverse cardiac events (MACE), but had experienced 2 episodes of anaphylaxis of an unknown cause. Patient 2 was diagnosed with non-obstructive HCM 9 years before enrolling the study. He had subclinical hypothyroidism, hypertension, and dislipemia. His medication included bisoprolol (2.5 mg/day), indapamide (1 tablet/day), and simvastatin (20 mg/day). He had one MACE (syncope) 20 years before enrolling the study, prior to his diagnosis of HCM. None of them had a smoking habit at the moment of the study, nor were they implantable cardioverter defibrillator carriers. Both patients had a low SCD risk according to the current guidelines and ESC risk calculator [13]. Both were informed about the possibility of participating in the study during their annual review with their cardiologist and gave their consent after being informed of the protocol, possible risks, and expected benefits. Further details of their baseline characteristics can be found in the pre-training values in the results section.

### 2.2. Experimental Design

**Figure 1:**
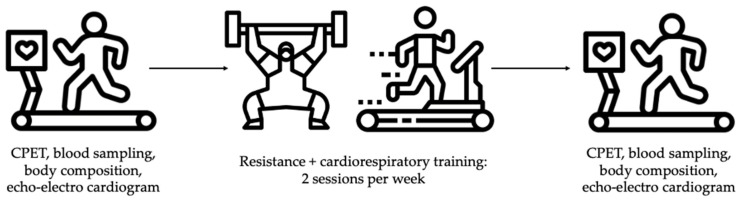
Experimental design.

The study was performed for a total duration of 14 weeks for each patient: a first week of assessments, followed by 12 weeks of concurrent resistance and cardiorespiratory training, and, finally, another week of assessments. During the first week, in addition to the initial evaluations in the hospital, a familiarization session with the material and exercises was carried out in the gym. After training, the patients were provided with a guide to continue this exercise autonomously following the same guidelines used during the intervention. A graphical diagram of the experimental design is shown in Figure 1 (icons contained in the figure were obtained from www.flaticon.es, accessed on 1 March 2024).

### 2.3. Testing Procedures

All assessments were conducted in the same facilities, utilizing identical materials and performed at consistent timings for both participants. During their initial visit, the patients were scheduled in the hospital at 15:00, with a recommendation to have their previous meal at least 3 h before, avoiding substantial food intake. Firstly, an echocardiogram was performed using a Phillips Epiq device (Amsterdam, The Netherlands). Subsequently, a blood sample of 8 mL was drawn from the left arm, followed by anthropometric measurements of the hip and waist using a standard tape measure, and a body composition analysis using electrical impedance: the patients were placed standing barefoot on an InBody 120 (Seoul, Republic of Korea) impedance scale, holding this position with raised arms a dual-entry handle that completed the impedance analysis with both foot entries. The patients were then positioned in supine decubitus for the placement of electrocardiogram electrodes (Cardioline ClickECG, Milan, Italy). In this position, readings of the resting electrocardiogram and blood pressure in the right arm (Cardioline) were obtained. An echocardiogram including standard 2D views, Doppler flow velocity, and tissue Doppler analysis was performed with a Philips iE33 Ultrasound System 795052 model (Amsterdam, The Netherlands). Following this, the patients were positioned on the treadmill (Runner-Run 7411, Cavezzo, Italy) and underwent functional assessment following the modified Bruce protocol [14]. Continuous monitoring during the test included gas consumption (Cortex Metalyzer 3B; Cortex Biophysik GmbH, Leipzig, Germany) and exercise electrocardiogram. The patients were encouraged to continue the test to maximum exertion. CPET was considered maximal when a respiratory exchange ratio (RER) of >1.10 was reached. Gas consumption data were used to determine the ventilatory thresholds and associated heart rate for each, and subsequently used to plan and monitor their cardiorespiratory training. Following the CPET, a second blood sample was extracted and both samples were, therefore, aliquoted and stored at −40 °C. All the assessments were repeated one week after finishing the training period.

Additionally, the determination of maximum strength for each resistance training exercise was carried out through the estimation of the 1 Repetition Maximum (1RM). This involved recording the weights and repetitions performed, and the patients were queried about their subjective effort perception according to Borg’s scale [15], indicating how many more repetitions they could have completed in each set. Epley’s formula [16], proven to be the most accurate for the exercises included in this study and beginner practitioners, was employed for the 1RM estimation [17]. Before effective sets (those considered as part of the training), the patients performed unloaded mobilization of the segments, replicating the movement involved for each exercise and an initial warm-up set of 10 repetitions with a low load (e.g., a load with which the patients could easily perform many repetitions). Calculations were conducted for all sets, and the highest estimation from the familiarization session and the first week of training was considered as the initial 1RM, while the highest estimation from weeks 11–12 of training was considered as the final 1RM. Since this was a pilot study involving two patients, statistical analyses were limited to direct comparisons of the pre- to post-training values.

### 2.4. Training Protocol

The training regimen comprised two weekly sessions over a span of 12 weeks, with a minimum 48 h separation between them. Each session incorporated a resistance training initial part followed by cardiorespiratory training, adhering to an order optimized for minimizing acute [18] and long-term [19] interference between the adaptations of both training modalities. Exercise selection excluded those involving trunk flexion or supine positions to mitigate the likelihood of orthostatic episodes. Six exercises, each targeting one muscle group—back, chest, shoulder, biceps, triceps, and legs—were conducted in each session, utilizing guided machines, pulleys, or free weights. The complete selection is detailed in Table 1.

For each exercise, a warm-up set (60–70% of the weight targeted for set 1) and three effective sets were performed across the 12 weeks, resulting in a total of 18 sets/day and 36 sets/week. A 2 min rest interval was established between sets [20]. Intensity was maintained within the 55–70% range of 1RM throughout the training period. The participants were instructed to choose a weight for each set, allowing them to complete between 16 and 24 repetitions, which corresponds to the specified relative intensities [21]. However, they were advised to execute approximately half of the potential repetitions with the selected loads, maintaining a moderate effort level (e.g., selecting a load they could complete ~20 repetitions with but performing only 8–10). This effort character entails a lower associated fatigue while allowing for greater adaptations in dynamic maximum strength compared to protocols employing high effort levels leading to muscular failure or near failure [22,23].

On the other hand, cardiorespiratory training was individually monitored and guided in all sessions using a heart rate monitor (Polar H10, Polar Electro, Kempele, Finland) simultaneously connected to the treadmill and a control watch for the researchers. The first session of each week focused on a pace slightly above the aerobic threshold (VT1), involving treadmill session from 25 to 50 min with no incline, adjusting the speed to achieve and maintain a heart rate of 5–10 bpm above that associated with the VT1 based on their CPET. The second session introduced fartlek-style interval training, featuring 2–6 min intervals 5–10 bpm below the second ventilatory threshold (VT2) intensity, interspersed with 1–2 min active rests at the VT1 pace, as previously proposed for this type of training [24]. The main segment duration progressively increased from 14 to 25 min in these sessions, with a 10-min warm-up and a 5-min cool-down at a comfortable, self-selected pace. Intensity adjustments were made by modifying the treadmill speed and incline, as in the CPET with the modified Bruce protocol. Full details of every cardiorespiratory training session can be found in Table A1.

## 3. Results

Both patients were overweighted at baseline and gained weight during the intervention, although a slight reduction in body fat was observed (−0.6% both), along with an average increase of 1 kg in muscular mass (Table 2). Patient 1 reduced his waist circumference by 3 cm, and patient 2 did so by 1 cm. Patient 1 also had a higher baseline waist-to-hip ratio (1.10), which reduced to a greater extent (−0.06) compared to patient 2 (−0.01).

Changes in supine and resting blood pressure varied between the two patients, as did differences in resting respiratory volumes and frequency, which were very subtle. The first consistent changes were observed in variables related to VT1, where, in both cases, a reduction in the heart rate required to achieve the same oxygen consumption was noted (−9 and −11 beats to reach 15 mL/kg/min pre- and post-training in both cases).

At VT2, both patients experienced an increase in oxygen consumption of nearly 0.5 L/min (0.36 and 0.43), translating to improvements of 3 and 6 mL/kg/min, respectively. Both patients also increased the heart rate associated with VT2 after training, thereby expanding the heart rate range between the first and second ventilatory thresholds. While ventilation showed minimal changes at VT1, increases of 13.5 and 20.0 L/min were observed at VT2, respectively.

Finally, VO_2_max increased by 4 mL/kg/min in both cases: +17% and +12% in predicted VO_2_max (Table 3), placing both patients above 100% in this parameter. Increased ventilation was also observed, similar to VT2, with values 19.0 and 14.3 L/min higher compared to the first CPET, resulting in an elevated respiratory frequency (+8 and +5 breaths/min). The maximum heart rate achieved increased notably in Patient 1 (+10 beats) and remained similar in Patient 2 (+1 beat). Regarding the recovery of heart rate after the first minute of active recovery, although changes were uneven between the two patients in the pre- and post-training comparisons, in both cases, the heart rate dropped by >18 beats after the first minute following CPET.

**Figure 2:**
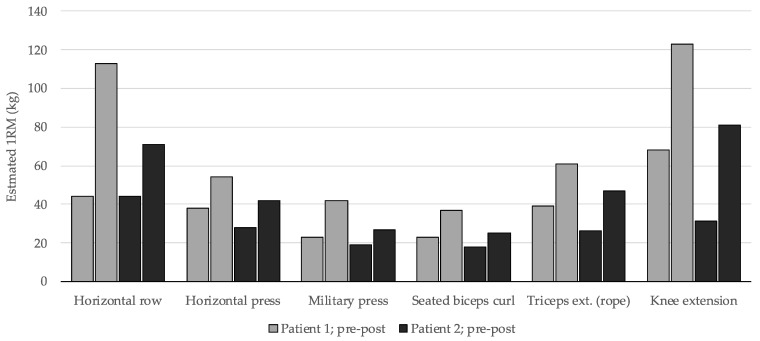
Pre-training (first column) and post-training (second column) estimated 1RM in the exercises of training session 1.

In the strength-related training outcomes, an enhancement in estimated 1RM was evident across all exercises and muscle groups in both patients. The increase in this variable ranged from 11% to 161%, with an average of a 62.2% improvement after the 12-week period (patient 1: 73%, patient 2: 56%). Figure 2 illustrates that the estimated 1RM for patient 1 was initially higher in five out of six exercises, yet their percentage increase was greater. Nevertheless, the strength improvement in patient 2 was also noteworthy. In both cases, the most substantial strength gains were observed in the back and leg exercises.

The electrocardiographic findings showed a decrease in the duration of the P wave, PQ interval, and QRS complex in Patient 1, while these remained unchanged in Patient 2 (Table 4). Both the QT and QTc interval were similar in the pre–post comparisons in both patients.

With regard to the resting echocardiographic findings (Table 5), the maximum wall thickness remained unchanged in both patients after training. However, the absolute and indexed left ventricular mass were augmented in both cases. The end-systolic diameter decreased by 0.2 and 0.3 cm, respectively, while the end-diastolic diameter behaved unevenly. The end-diastolic volumes also showed disparities between the patients, while the end-systolic volume was slightly augmented. The left ventricular ejection fraction slightly diminished in both patients, but stayed at around 70%. In patient 2, reductions in left atrial diameter and volume were observed. Patient 2 also exhibited decreases in Vmax E, lateral E, medial E, and medial and lateral E/E′ ratios, while patient 1 only in medial and lateral E/E′.

The blood analysis (Table 6) showed an increase in serum glucose, although changes must be interpreted cautiously, since blood sampling was performed in a non-fasted state. The serum minerals remained mostly unchanged or with slight variations. Cardiac markers such as cardiac troponin T and NT-proBNP showed uneven changes: both markers decreased in one patient’s higher baseline values, while slightly increased in the other. Finally, changes in hormones were more consistent between the patients, with a decrease in serum testosterone and increments in basal cortisol and TSH. No adverse events nor complications of any type occurred during training. Both patients attended 100% of the sessions.

## 4. Discussion

One of the primary objectives of this intervention was to assess the feasibility of the proposed concurrent training protocol, with the aim of replicating it in a larger cohort of patients. Both the CPET and all training sessions were devoid of adverse events and complications, thereby establishing the feasibility of the training protocol.

Regarding previous investigations, in that by Saberi et al. [8], patients underwent 16 weeks of cardiorespiratory training with 2–3 sessions per week at an intensity of 60–70% of heart rate reserve (HRR). The duration progressed from 20 to 60 min, adding 5–10 min in each session. On the other hand, Klempfner et al. [9] also prescribed exercise based on HRR, starting with a 10 min warm-up at 40–50% of the HRR and then completing a 60 min session with an intensity increasing from 50–60% of the HRR to 65–85%. The authors stated that two sessions were conducted weekly until completing an average of 41 h of training, which suggests an approximate duration of ~20 weeks, although they did not explicitly state this. Wassestrum and colleagues [7] also programmed training sessions based on HRR, but the time between pre- and post-training assessments was very uneven and up to more than three years, which may not precisely identify the changes elicited by the training protocol (Table 7).

Regarding cardiorespiratory training, the most recent trial [10] included significant improvements in intensity control compared to previous ones, precisely defining training zones based on the maximum heart rate and heart rate associated with VT2 in a previous CPET. This intervention was also the first to incorporate high-intensity intervals at 90–95% of peak heart rate. During their study, the authors did not record any serious adverse events. However, to date, only one intervention has considered strength training in its protocol [11], in addition to cardiorespiratory training. In this intervention, participants engaged in three sessions per week for 18 months, cycling at 60–80% of their VO_2_max and subsequently performing upper limb pushing and pulling exercises, lower limb extensions, trunk flexion, and three unspecified stretching exercises. The intensity in these strength exercises was set at 65% of each subject’s 1RM, although they did not specify how this was calculated or provide progression details for the loads used or the number of repetitions performed.

Previous studies have demonstrated that the optimal distribution of concurrent training to maximize benefits and minimize interference in acute and long-term adaptations is to conduct strength training at the beginning of the session, followed by cardiorespiratory exercise [19,25]. Moreover, it has been observed that the benefits of concurrent training in strength and cardiorespiratory aspects surpass those observed with either modality performed exclusively, especially in sedentary, beginners, and intermediate individuals [12]. Finally, recent advancements in exercise physiology have underscored the role of skeletal muscle as an endocrine tissue in cardiovascular risk prevention [26]. Skeletal muscle releases numerous myokines, particularly during exercise-induced contractions, with potential positive impacts on HCM progression, such as reducing pro-inflammatory status, mitigating vascular stenosis and atherosclerotic processes, or promoting revascularization of ischemic tissue [27,28].

When interpreting changes in body composition, caution should be warranted, as the subjects’ diet was not controlled during the study. Nevertheless, the observed average gain of 1 kg in muscle mass coupled with a 0.6% reduction in body fat percentage provide encouraging data. A previous study examined the effect of cardiorespiratory training on body composition, wherein no changes were observed in the amount of lean mass or body fat among the subjects [10]. Putting together these findings with those observed in our study, it appears that incorporating resistance training alongside the traditionally employed cardiovascular training could prove to be an effective strategy for enhancing body composition. Furthermore, the decrease in the waist-to-hip ratio also suggests an improvement in body composition, which is beneficial for overall health [11]. It is essential to note that both patients were overweight (with Patient 1 classified as obese), a factor previously linked in studies with impaired functional capacity and potential negative effects on symptomatic status and quality of life [29,30]. Future studies should incorporate dietary control and/or interventions aimed at weight loss.

In the realm of exercise testing, numerous derived parameters have shown a significant association with the prognosis of disease progression, mortality, and the occurrence of medium- to long-term events [31]. These parameters encompass variables related to functional capacity (VO_2_max, % of predicted VO_2_max, and VO_2_ at VT2), ventilatory efficiency (V′E/V′CO_2_), and heart rate (peak HR and post-exercise heart rate recovery).

Firstly, improvements in VO_2_max have been linked to a reduced risk of mortality and events [32,33,34,35,36]. The benefits observed in our intervention are promising, with a 4 mL/kg/min increase in VO_2_max in both cases. The latter aligns with findings from a previous meta-analysis conducted by our group, encompassing the prior exercise interventions [6]. Additionally, Finocchiaro et al. reported a 48% reduction in the composite end-point risk for every 5 mL/kg/min increase in VO_2_max [37]. Notably, the composite endpoints included not only standard events, but also functional deterioration leading to septal reduction therapy. Thus, the functional capacity improvement achieved with the proposed training not only decreases the risk of mortality and severe events, but also the need for invasive therapies.

This absolute increase in VO_2_max concurrently implies an improvement in the % of the predicted VO_2_max, a value associated with a better prognosis and increased survival [34,35,36,37,38]. The respective 17% and 12% increases placed both patients above 100% of their predicted values.

Another parameter of interest due to its relationship with long-term prognosis improvement is oxygen consumption at the anaerobic threshold [31]. In our patients, notable increases in this variable were also observed (3 and 6 mL/kg/min). Studies have reported a roughly 20% decrease in the risk of events and sudden death with improvements in this parameter [32,34,35], emphasizing the benefits of the proposed training program.

On the other hand, ventilatory efficiency, as assessed by the V′E/V′CO_2_ ratio at the moment of VO_2_max, has been correlated with a poorer prognosis [32], especially when exceeding a ratio of 31 [34] and 34 [37]. In our study, both patients had lower values before and after training, although a slight increase was observed in both cases. However, the recorded ratios are not alarming, as they remain below the aforementioned reference thresholds.

Finally, the recorded maximum heart rate has been linked to a 23% decrease in the risk of composite endpoint for every 10 beats increase [37]. In this context, Patient 1 increased his maximum heart rate by 10 beats after training, representing another positive indicator. In addition to the maximum attained, the recovery of heart rate following the completion of CPET is also employed to assess cardiac function. In this regard, Masri et al. demonstrated a 52% reduction in the risk of reaching a composite endpoint in patients who exhibited a reduction of at least 18 bpm in the first minute post-exercise [38]. It is crucial to note that the CPET protocol they utilized concluded without a recovery phase, whereas ours included one. Nevertheless, both of our patients recovered more than 18 bpm in the first minute after the training period. In the case of Patient 2, during the initial CPET, only a 10 bpm recovery was observed, underscoring the beneficial impact of the training in this aspect as well. Furthermore, the increment (right-shift) observed in the heart rate associated with VT2 and left-shift in that of VT1 increase training possibilities through broadening the inter-threshold range of viable exercise intensities.

Regarding strength adaptations, the long-term implications concerning the prognosis and progression of hypertrophic cardiomyopathy remain unknown, as our study represents the first to incorporate such a strength training protocol. Notably, enhancements in strength and the augmentation of musculoskeletal tissue are linked to health benefits, including a reduction in cardiovascular risk [26,27]. Consequently, it is expected that the strength improvements achieved through the proposed training regimen will exert a positive impact on the health and quality of life of the patients.

In relation to the electrocardiographic findings, the decrease in the P wave duration and PQ interval observed in Patient 1 may be explained by the presence of COPD. Other than that, the training protocol seemed to have no effect on the electrocardiographic parameters. However, further observation is needed, especially in patients with comorbidities affecting the lungs or a smoking history.

Echocardiographic features demonstrated signs of left ventricular remodeling, despite there only being two patients. As the left ventricular diameter is one of the more reliable measurements, it increased by 0.6 and 0.3 cm with minor variations in systolic function. These findings are consistent with those of the most recent protocol, where both moderate- and high-intensity cardiorespiratory training showed a significant 0.3 cm increase in this parameter [10]. Measurements of left ventricular volumes in patients with hypertrophic cardiomyopathy from echocardiography are subject to errors related to the assumption of the Simpson method in asymmetric hypertrophy. As expected, there was no change in wall thickness. Changes in strain parameters and left atrial dimensions might seem too inconsistent and should be taken cautiously. Although these changes have to be considered cautiously given the preliminary nature of the results, available data suggest better outcomes with the proposed program compared to that referred by previous studies, where no changes in echocardiographic findings were observed [7,8,11]. More interestingly, with the limitation of a pilot study, the exercise program seemed to have a positive impact on diastolic function, with a consistent reduction in the E/e’ ratios.

Cardiac troponin T, which is a biomarker of myocardial damage, remained within normal range, which is a reassuring finding of the short-term safety of the training, while NT-proBNP showed a mild increase in one of the patients and a more relevant decrease the other participant. An increase in this biomarker might be expected with exercise in patients with ventricular dysfunction. However, the observed decrease in patient 2 could suggest that the exercise did not impose a significant overload on the ventricle, or it could even indicate an improvement in ventricular function or the heart’s ability to handle physical stress over time, possibly as a result of adapted and controlled physical training. Variations of <100 pg/mL can be considered as day-to-day changes in stable cardiomyopathy patients. An evaluation of a larger number of patients would establish the real impact of training on NT-proBNP values, since previous studies have reported similar values at post-training [8,11].

Concerning hormones, the acute increase in circulating testosterone levels is noteworthy following exercise [39], whereas chronic exposure to training does not imply an elevation in resting concentration [40]. What does persist over a longer period (24–48h) is the mRNA and protein content in androgen receptors, predisposing the organism to greater anabolic adaptation in response to exercise [41]. These findings align with the observed muscle mass increase in the subjects of this pilot study: the slight decrease in baseline testosterone could be attributed to other factors such as age, and may not be a consequence of exercise. In this context, the circulating levels of steroid hormones are contingent upon the individual rate of biosynthesis, the presence of binding proteins in circulation, and modulation through the rate of the hormonal metabolic clearance process [42]. As for changes in basal growth hormones, it is imperative to observe this response in a larger number of patients, as disparate results were found in the two subjects of this pilot study: while patient 1 experienced an increase in concentration, patient 2 exhibited a slight reduction. Nevertheless, the elevation of serum GH is evident within the first 10–20 min of exercise and persists up to 60 min post-exercise, but not chronically [43,44]. Regarding cortisol, despite a slight increase in the baseline values in both patients, both pre- and post-exercise levels were within the expected range. Moreover, it is acknowledged that cortisol levels are significantly influenced by factors such as stress levels, prior meals, or circadian rhythm [45]. Therefore, although we will continue to monitor the results in a larger cohort, the subtle changes in this hormone do not seem to be affected by the proposed training and/or clinically relevant. Finally, studies linking exercise with TSH concentration have revealed an inverse relationship between the degree of physical activity and hormone levels [46] or, at least, no changes after 12 weeks of training [47]. Consequently, the increase in the TSH concentration in patient 2 along with normal levels of free T4 may have been linked to the presence of subclinical hypothyroidism [47].

## 5. Study Limitations

However, this study is not without limitations that must be addressed. The first of these concerns the small number of included patients: while the results are promising, it is still necessary to replicate this protocol in a larger cohort to validate them. Additionally, there are considerations regarding duration and follow-up. Regarding the duration, it would be interesting to observe the long-term effects of incorporating the training as part of the patients’ habitual lifestyle (for months and years). Concerning follow-up, it would be beneficial to include an assessment after a longer post-training period to determine if improvements in functional capacity, strength, and cardiac function are sustained and for how long.

## 6. Conclusions

Training in patients with HCM has proven effective in improving functional capacity and variables derived from CPET and other cardiac parameters associated with a better prognosis. The concurrent resistance and cardiopulmonary training protocol proposed in this pilot study appeared to achieve similar benefits in functional capacity as observed in previous studies, albeit in a shorter timeframe and with fewer sessions. Moreover, it may further improve other parameters related to functional class and medium-to-long-term prognosis, such as body composition, diastolic function, and reversal of adverse cardiac remodeling. The positive results obtained encourage the replication of the study in a larger patient cohort to verify the adaptations achieved.

## Figures and Tables

**Table 1:** Exercises included in resistance training.

Muscular Group	Session 1	Session 2
Back	Seated horizontal row	Prone-grip pull-downs
Chest	Seated horizontal press	Seated machine chest-flys
Shoulders	Seated military press	Lateral raises w/dumbbell
Biceps	Seated dumbbell curls	Standing dumbbell curls
Triceps	High pulley ext. (w/rope)	High pulley ext. (w/bar)
Legs	Seating knee extensions	Seated horizontal leg press

**Table 2:** Anthropometric and body composition characteristics.

		Patient 1			Patient 2	
	Pre	Post	Change	Pre	Post	Change
Height (cm)	170	170	-	161	161	-
Weight (kg)	95.7	97.1	1.4	67.8	69.9	2.1
BMI (kg/m^2^)	33.1	33.6	0.5	26.2	27	0.8
Muscular mass (kg)	36.3	37.1	0.8	25.6	26.8	1.2
Body fat (%)	32.8	32.2	−0.6	31.9	31.3	−0.6
Fat mass (kg)	31.4	31.2	−0.2	21.6	21.9	0.3
Waist circumference (cm)	110	107	−3	87	86	−1
Hip circumference (cm)	100	103	3	93	93	-
Waist-to-hip ratio	1.10	1.04	−0.06	0.94	0.93	−0.01

BMI: body mass index.

**Table 3:** Cardiopulmonary exercise test.

	Patient 1			Patient 2		
	Pre	Post	Change	Pre	Post	Change
Supine heart rate	63	63	-	68	60	−8
Supine systolic BP	130	162	32	181	142	−39
Supine diastolic BP	89	92	3	90	87	−3
Resting heart rate	70	65	−5	74	65	−9
Resting systolic BP	124	151	27	148	163	15
Resting diastolic BP	88	94	6	84	95	11
**(a) Resting**						
VO_2_ (L/min)	0.48	0.41	−0.07	0.38	0.39	0.01
VO_2_/kg (mL/kg/min)	5	4	−1	6	6	-
V′E (L/min)	16.7	13.9	−2.8	13.2	11.3	−1.9
V′E/V′CO_2_	32.5	33.1	0.6	32.5	28.5	−4.0
Breathing frequency	19	17	−2	13	17	4
**(b) Ventilatory threshold 1**						
VO_2_ (L/min)	1.45	1.51	0.06	0.99	1.01	0.02
VO_2_/kg (mL/kg/min)	15	15	-	15	15	-
VO_2_/kg (% max)	60.0	51.7	−8.3	46.9	41.7	−5.2
Heart rate (bpm)	101	92	−9	97	86	−11
Heart rate (% max)	72.1	61.3	−10.8	69.8	61.4	−8.4
V′E (L/min)	35.1	35.3	0.2	26.4	24.6	−1.8
V′E/V′CO_2_	26.3	28.4	2.1	29.0	29.6	0.6
Breathing frequency	22	23	1	20	24	4
**(c) Ventilatory threshold 2**						
VO_2_ (L/min)	2.36	2.73	0.37	1.69	2.12	0.43
VO_2_/kg (mL/kg/min)	25	28	3	25	31	6
VO_2_/kg (% max)	100	96.5	−3.5	78.1	86.1	8.0
Heart rate (bpm)	140	146	6	119	128	9
Heart rate (% max)	100	97.3	−2.7	85.6	91.4	5.8
V′E (L/min)	68.9	82.4	13.5	44.1	64.1	20.0
V′E/V′CO_2_	24.1	25.3	1.2	26.2	28	1.8
Breathing frequency	29	34	5	22	32	10
**(d) Maximum VO_2_**						
VO_2_max (L/min)	2.36	2.78	0.42	2.18	2.41	0.23
VO_2_max (mL/kg/min)	25	29	4	32	36	4
VO_2_max (% pred.)	95	112	17	109	121	12
Heart rate (bpm)	140	150	10	139	140	1
Heart rate recovery 1′ (bpm)	36	20	−16	10	19	9
Respiratory exchange ratio	1.14	1.16	0.02	1.14	1.13	0.01
V′E (L/min)	68.4	87.4	19.0	71.0	85.3	14.3
V′E/V′CO_2_	24.2	25.4	1.2	28.6	29.3	0.7
Breathing frequency	29	37	8	32	37	5
Max. slope (%)	14	14	-	14	14	-
Max. speed (km/h)	5.3	5.6	0.3	5.4	5.6	0.2
Test duration (min)	8′21″	8′56″	0′35″	8′31″	9′27″	0′56″

Heart rate recovery after 1′ shows the difference between max HR and HR 1′ after the completion of CPET, during the walking recovery phase. BP: blood pressure.

**Table 4:** Electrocardiographic findings at rest.

		Patient 1			Patient 2	
	Pre	Post	Change	Pre	Post	Change
P wave (ms)	156	98	−58	116	114	−2
PQ interval (ms)	174	154	−20	120	120	-
QRS complex (ms)	110	90	−20	98	98	-
QT interval (ms)	396	396	-	438	448	10
QTc interval (ms)	402	401	−1	453	450	−3

**Table 5:** Echocardiography.

	Patient 1			Patient 2		
	Pre	Post	Change	Pre	Post	Change
MWT (mm)	17	17	-	15	15	-
LVEDD (cm)	4.4	5.0	−0.6	4.8	5.1	0.3
LVESD (cm)	3.0	2.8	−0.2	3.5	3.2	−0.3
LVEDV 4C (mL)	134.1	114.3	−19.8	68.3	86.5	18.2
LVESV 4C (mL)	29.5	32.9	3.4	18.5	27.4	8.9
Indexed LVEDV (mL/m^2^)	63.7	55.0	−8.7	39.8	49.7	9.9
Indexed LVESV (mL/m^2^)	14.0	15.8	1.8	10.8	15.8	5.0
LV mass (g)	220.5	276.5	56.0	171.3	197.1	27.8
Indexed LV mass (g/m^2^)	104.7	133.0	28.3	99.9	113.3	13.4
FS (%)	31.5	43.6	12.1	26.5	36.6	10.1
PWT (mm)	10.0	10.4	0.4	8.1	10.1	2.0
LVEF 4C (%)	78.0	71.2	−6.8	72.9	68.3	−4.6
TAPSE (cm)	2.10	2.48	0.38	2.10	2.42	0.32
LA SGL (%)	31.0	27.9	−3.1	23.8	17.6	−6.2
LA diameter (cm)	4.8	4.8	-	4.5	4.0	−0.5
LA volume 4C (mL)	88.9	89.0	0.1	101.9	89.0	−12.9
Indexed LA volume (mL/m^2^)	40.5	42.8	2.3	48.4	51.2	2.8
Vmax E (cm/s)	97.0	87.3	−9.7	83.4	63.5	−19.9
Vmax A (cm/s)	105.3	103.8	−1.5	38.3	35.9	2.4
E/A	0.92	0.84	−0.08	2.20	1.77	0.43
Vmax medial E′ (cm/s)	4.5	6.3	1.8	6.7	6.8	−0.1
Vmax lateral E′ (cm/s)	6.5	9.1	2.6	7.3	5.9	−1.4
Medial E/E′	21.3	13.9	−7.4	12.5	9.4	−3.1
Lateral E/E′	15.0	9.6	−5.4	11.4	5.9	−5.5

FS (%): percentage of fractional shortening, LA: left atrial, LV: left ventricular, LVEDD: left ventricular end-diastolic diameter, LVEDV: left ventricular end-diastolic volume, LVESD: left ventricular end-systolic diameter, LVESV: left ventricular end-systolic volume, MWT: maximum wall thickness, PWT: posterior wall thickness, and TAPSE: tricuspid annular plane systolic excursion.

**Table 6:** Blood analysis.

		Patient 1			Patient 2	
	Pre	Post	Change	Pre	Post	Change
Glucose (mg/dL)	83	90	7	85	96	11
Urea (mg/dL)	33	37	4	38	44	6
Sodium (mEq/L)	139	139	-	138	138	-
Potassium (mEq/L)	4.7	4.1	−0.6	4.0	3.8	−0.2
Chlorine (mEq/L)	101	103	2	98	97	−1
Cardiac troponin T (pg/mL)	17	14	−3	6	8	2
NT-proBNP (pg/mL)	144	243	99	514	282	−232
Interleukin 6	3.2	2.8	−0.4	1.5	1.5	-
Testosterone (ng/mL)	3.11	2.12	−0.99	5.42	5.03	−0.39
Basal cortisol (mcg/dL)	6.9	7.3	0.4	9.2	11.6	2.4
Basal GH (ng/mL)	0.03	1.71	1.68	0.50	0.16	−0.34
TSH (uIU/mL)	2.95	3.78	0.83	4.43	7.29	2.86

GH: growth hormone, NT-proBNP: N-terminal pro B-type natriuretic peptide, TSH: thyroid-stimulant hormone.

**Table 7:** Methodological aspects of previous investigations including exercise protocols in HCM patients.

Author, Year	Duration, Sessions	Cardiorespiratory Training	Resistance Training	Additional Measures
Klempfner, 2015 [9]	2 sessions/week (41 h in total) no more details on duration/schedule	50 to 85% of HRR and 13 to 15 points of RPE, progression not explained	Not included	Holter, physical examination and echocardiography
Saberi, 2017 [8]	16 weeks 4–7 sessions/week	60 to 70% of HRR and 12 to 14 points of RPE, progression explained	Not included	Electrocardiogram, echocardiography, blood tests, CMR, genetic testing
Wasserstrum, 2019 [7]	3 to 4 months 2 sessions/week	60 to 70% of HRR and 13 points of RPE, progression not explained	Not included	Electrocardiogram, echocardiography
Limongelli, 2021 [11]	18 months 3 sessions/week	60 to 80% of VO_2_max, progression not explained	Exercises poorly defined. Intensity at 65% of 1RM but neither 1RM estimation nor volume, rest, etc. explained	Holter, physical exam, CMR, blood tests, echo-electro cardiography
MacNamara, 2023 [10]	5 months 3–5 sessions/week	Two groups with different %HR based on peak HR and MSS from CPET; progression well-explained	Not included	Echocardiography, body composition, blood tests

Progression refers to weekly changes in volume, intensity (value and changes/constant intensity) and duration of each bout of exercise. CMR: cardiac magnetic resonance, CPET: cardiopulmonary exercise test, HRR: heart rate reserve, MSS: maximum steady state, RPE: rate of perceived exertion, and 1RM: 1-repetition maximum.

## Data Availability

Data are available upon reasonable request to corresponding author.

## Appendix A

**Table A1:** Cardiorespiratory training sessions.

Week	Session 1	Session 2
Week 1	25 min at VT1 + 5/10 bpm	7 × (2 min pace/1 min rec)
Week 2	25 min at VT1 + 5/10 bpm	6 × (3 min pace/1 min rec)
Week 3	30 min at VT1 + 5/10 bpm	5 × (4 min pace/2 min rec)
Week 4	30 min at VT1 + 5/10 bpm	4 × (5 min pace/2 min rec)
Week 5	35 min at VT1 + 5/10 bpm	7 × (3 min pace/1 min rec)
Week 6	35 min at VT1 + 5/10 bpm	5 × (4.5 min pace/1.5 min rec)
Week 7	40 min at VT1 + 5/10 bpm	4 × (5 min pace/2 min rec)
Week 8	40 min at VT1 + 5/10 bpm	6 × (4 min pace/1.5 min rec)
Week 9	45 min at VT1 + 5/10 bpm	4 × (6 min pace/2 min rec)
Week 10	45 min at VT1 + 5/10 bpm	5 × (5 min pace/2 min rec)
Week 11	50 min at VT1 + 5/10 bpm	5 × (5 min pace/2 min rec)
Week 12	50 min at VT1 + 5/10 bpm	5 × (4 min pace/1.5 min rec)

Bpm: beats per minute, VT1: first ventilatory threshold. “Pace” refers to the most intense bouts of the training session, aiming at an intensity 5/10 bpm below the heart rate associated to the second ventilatory threshold as extracted from the CPET. “Rec”: recovery; refers to the recovery lapse interspersed with the pace bouts. These were performed at the same intensity (speed and incline) of session 1.
